# Feasibility of clinical implementation and acceptability of patient-reported screening for chemotherapy-induced peripheral neuropathy (CIPN)

**DOI:** 10.1007/s00520-026-11296-4

**Published:** 2026-10-08

**Authors:** Alice Treloar, Peter Grimison, Victoria Choi, Jed Young, Katelin Mayer, Tiffany Li, Frances Boyle, Tracy King, David Mizrahi, Lisa G. Horvath, David Goldstein, Susanna B. Park

**Affiliations:** 1 Brain and Mind Centre, The University of Sydney, Sydney, NSW Australia; 2 Chris O’Brien Lifehouse, Sydney, NSW Australia; 3 Sydney Medical School, The University of Sydney, Sydney, NSW Australia; 4 Faculty of Medicine and Health, The University of Sydney, Sydney, NSW Australia; 5 Prince of Wales Hospital, Sydney, NSW Australia; 6 Patricia Ritchie Centre for Cancer Care and Research, Mater Hospital, Sydney, NSW Australia; 7 Institute of Haematology, Royal Prince Alfred Hospital, Sydney, NSW Australia; 8 Faculty of Medicine and Health, Susan Wakil School of Nursing and Midwifery, Cancer Care Research Unit, The University of Sydney, Sydney, NSW Australia; 9 The Daffodil Centre, The University of Sydney, and Cancer Council NSW, Sydney, NSW Australia; 10 University of New South Wales, Sydney, NSW Australia; 11 School of Medical Sciences, Faculty of Medicine and Health, The University of Sydney, Sydney, NSW Australia

**Keywords:** Chemotherapy induced peripheral neuropathy, Screening, Cancer, Quality of life, CIPN

## Abstract

**Purpose:**

Chemotherapy-induced peripheral neuropathy (CIPN) is a major side-effect of cancer treatment, leading to early treatment cessation and long-lasting disability. This project aimed to determine acceptability and barriers of routine CIPN screening.

**Methods:**

In a prospective sample across two sites, patients scheduled for treatment or treated with neurotoxic therapy were eligible to complete CIPN screening at any cycle. Three brief patient-reported outcome screening tools (NCI-PRO-CTCAE, PNQ, and EviQ) and a short feasibility questionnaire were deployed (termed *CIPN-Screen)* during appointments (Centre 1) or via automated text messages (Centre 2).

**Results:**

Seven hundred fifty-five screens were undertaken in 159 participants between March 2022 and November 2023, with 109 at Centre 1 (median[IQR] 1[1]screen) and 646 at Centre 2 (median[IQR] 8[6]screens). Automatic text message reminders at Centre 2 enabled longitudinal assessments to be collected. 426 screens (56.4%) identified CIPN symptoms with 138 (18.3%) moderate-to-severe. Most respondents reported that the CIPN-Screen was easy to complete (92.0%, *n* = 671), easy to understand (91.6%, *n* = 668), and helpful to describe symptoms (85.6%, *n* = 624). Acceptability did not differ by age, sex, or chemotherapy type; however, acceptability did differ according to CIPN symptom severity (*P* = 0.0002). There were significant differences in CIPN symptom score over time (*P* < 0.0001, *n* = 52), indicating that the CIPN-Screen detected symptom evolution over time.

**Conclusion:**

Questionnaire-based CIPN screening is feasible and assists in symptom profiling for patients treated with neurotoxic chemotherapy.

**Implications for cancer survivors:**

CIPN is an important toxicity with lasting effects for cancer survivors. Identifying better approaches to patient-relevant CIPN screening will enable improved care for cancer survivors.

**Supplementary Information:**

The online version contains supplementary material available at https://doi.org/10.1007/s00520-026-11296-4.

## Introduction

Chemotherapy-induced peripheral neuropathy (CIPN) is a potential adverse effect of cancer therapy with neurotoxic agents including taxanes, platinum compounds, vinca alkaloids, proteasome inhibitors and antibody–drug conjugates. CIPN produces symptoms of dysesthesia, paresthesia, pain, numbness, or burning in the hands and feet during chemotherapy treatment [1]. These symptoms can persist long-term and lead to functional disability, impairing fine motor ability, balance and quality of life, affecting >70% of cancer survivors treated with neurotoxic therapies [2].

CIPN symptoms can influence treatment, leading to dose reduction or early cessation. This affects planned treatment delivery and may interfere with clinical outcomes [3, 4], underscoring the need for clinical screening. Appropriate dose reduction or premature cessation of chemotherapy treatment in patients who develop significant neuropathy and/or functional impairment is recommended by consensus guidelines to prevent chronic CIPN [5], however, relies on accurate identification of toxicity. Clinical frameworks recommend screening for CIPN at baseline and prior to each chemotherapy treatment [6, 7]. However, barriers to uptake of these recommendations include the paucity of guidance demonstrating an optimal screening procedure [6], and logistic barriers relating to clinic time constraints and limited resource allocation.

Patient-reported outcome measures (PROMs) have demonstrated ability to identify CIPN sensitively and early [8–10]. Short patient-reported CIPN screening tools are correlated with longer patient-reported outcome measures and display good discriminant properties to identify patients at risk of experiencing neuropathy [10]. However, the uptake of these tools in routine practice has been slow and there is a lack of evidence regarding how to effectively support uptake of routine CIPN screening. Accordingly, this study aimed to investigate the acceptability of implementing enhanced screening for CIPN into routine clinical care from both a patient and clinician perspective, using brief patient-reported outcome screening tools and exploring automated electronic methods to overcome logistic barriers.

## Methods

### Participants

Participants aged ≥18 who were receiving or scheduled to receive neurotoxic chemotherapy (including platinum, taxane and anti-microtubule agents, vinca alkaloid, and bortezomib based regimens) were eligible to participate and recruited from two oncology centres in Sydney, Australia between March 2022 and November 2023. Participants could enrol before or after the commencement of neurotoxic chemotherapy. The study was approved by St. Vincent’s Hospital Human Research Ethics Committee (2020/ETH02551), with informed consent obtained from each participant.

### Neuropathy screening instruments

All participants completed a battery of CIPN screening instruments (termed CIPN-Screen) to examine self-reported neuropathy symptoms associated with chemotherapy treatment. CIPN-Screen consisted of three patient-reported outcome measure screening tools: the 2-item National Cancer Institute Patient Reported Outcomes version of the Common Terminology Criteria for Adverse Events (NCI-PRO-CTCAE) sensory neuropathy subscale [11], the 2-item Patient Neurotoxicity Questionnaire (PNQ) [12], and the 11-item Cancer Institute NSW EviQ online neurotoxicity instrument [13]. Participants who reported CIPN symptoms of any severity on screening tools were invited to complete the longer 20-item questionnaire, European Organization for Research and Treatment of Cancer Quality of Life Questionnaire Chemotherapy Induced Peripheral Neuropathy (EORTC-QLQ-CIPN-20) [14]. Details provided in Supplementary methods.

### Study procedures

At Centre 1, clinical staff approached eligible participants whilst attending chemotherapy treatment visits and offered a QR code to access the study material on their device (via REDCap) or a paper copy of the study material. Screens at Centre 1 relied on clinical staff approaching the participants at each treatment visit and were predominantly completed at one time point; however, participants could complete the CIPN-Screen during multiple treatment visits, with no upper limit on study participation. 

At Centre 2, clinical staff approached eligible participants while attending clinical appointments and offered to access the study material (via Qualtrics) on their device. Once participants were enrolled in the study, they were sent an automatic text message reminder to complete the CIPN-Screen at every chemotherapy treatment appointment without further input from clinical staff.

### CIPN-Screen acceptability

Following each screening, participants answered three acceptability questions regarding whether the CIPN-Screen was easy to complete, easy to understand, and helpful in describing their symptoms using a 5-point Likert scale from strongly disagree (0) to strongly agree (4) (Supplementary methods). The completion rate was defined as the proportion of submitted responses that had completed all three screening tools (NCI-PRO-CTCAE, PNQ, and EviQ). Acceptability was assessed in terms of the percentage of completed questions.

To assess the clinician perspectives of the CIPN-Screen implementation, 31 medical and nursing clinicians as well as allied health personnel completed the adapted Organisational Readiness for Implementing Change (ORIC) questionnaire [15]. Details are provided in Supplementary methods.

### Statistical analysis

All analysis was completed using GraphPad Prism, Version 10.2.3, GraphPad Software. Incomplete responses were omitted from analysis. Median (interquartile range) or mean (standard deviation) were used to describe data. Interquartile range (IQR) was reported as a single value, representing the difference between the 75th percentile and the 25th percentile. Characteristics were compared using Mann–Whitney tests or Chi-Square tests as appropriate. A composite score for the three acceptability questions was created by summing individual scores (score range 3–15), with higher scores representing greater acceptability. Chi-square tests were used to compare responses between centres and responses over time between screen 1 and 6 (Centre 2). Mann–Whitney *U* tests were used to compare scores by age, sex and chemotherapy type (Platinum vs Taxane ± Carboplatin). Kruskal–Wallis test with Dunn’s post hoc test was used to compare CIPN severity across acceptability questions, examine change in CIPN symptoms over time (screens 1–3) and compare CIPN-20 scores within NCI-PRO-CTCAE grades. Friedman’s test was used to compare change in CIPN symptoms across screens 1 to 7. Spearman’s correlation was used to compare the association between screening tools and association between subscales of the ORIC questionnaire. The interpretation of correlation strengths was 0–0.3 = negligible; 0.3–0.5 = weak; 0.5–0.7 = moderate; > 0.7 = strong [16].

## Results

### Cohort characteristics

In total, 159 participants were recruited for the study (Centre 1 *n* = 75; Centre 2 *n* = 84). There were significantly more females at Centre 2 (*P* < 0.0001; Table 1), and the median [IQR] age was younger (49 [14] years) compared to Centre 1 (61(23) years, *P* < 0.0001). At Centre 1, platinum was the most common neurotoxic chemotherapy (46.7%, *n* = 35; Table 1), and the most common cancer type was colorectal or other gastrointestinal cancers (36.0%, *n* = 27). At Centre 2, most participants were treated with taxanes (72.6%, *n* = 61) for breast cancer (77.4%, *n* = 65).

**Table 1:** Demographics, cancer and chemotherapy type

	Centre 1	Centre 2	*P* value
**Number of participants**	75	84	
**Age, years (median, IQR)**	61 [23]	49 [14]	*P* < 0.0001
**Gender [*n* (%)]**			
Female	38 (51.0%)	74 (88.1%)	*P* < 0.0001
Male	37 (49.0%)	10 (11.9%)	
**Cancer types [*n* (%)]**			*P* < 0.0001
Breast	21 (28.0%)	65 (77.4%)	
Colorectal	14 (18.7%)	2 (2.4%)	
Other gastrointestinal	13 (17.3%)	3 (3.6%)	
Gynecological	-	5 (6.0%)	
Haematological	10 (13.3%)	-	
Sarcoma	3 (4.0%)	9 (10.7%)	
Other	14 (18.7%)	-	
**Neurotoxic chemotherapy types [*n* (%)]**			
Bortezomib	6 (8.0%)	-	
Platinum	35 (46.7%)	4 (4.8%)	
Platinum and taxane	8 (10.7%)	17 (20.2%)	
Taxane	17 (22.7%)	61 (72.6%)	
Other	9 (12.0%)	2 (2.4%)	

Centre 1 Other cancer types include lung (*n *= 1), testicular (*n *= 2) and urothelial (*n *= 2), prostate (*n *= 4), and head and neck (*n *= 5). Other chemotherapy types include Carboplatin (*n *= 3), Vincristine (*n *= 3), Trastuzumab emtansine (*n *= 1), Eribulin (*n *= 1), and Carboplatin & Cabazitaxel (*n *= 1) for Centre 1 and Etoposide & Ifosfamide & mesna (*n *= 1) and nab−paclitaxel (*n *= 1) for Centre 2. Platinum=Cisplatin, Oxaliplatin; Platinum/Taxane=Docetaxal and Carboplatin, Paclitaxel and Carboplatin; Taxane=Docetaxel, Paclitaxel

### Overview of CIPN-Screen

Overall, 755 screens were undertaken amongst 159 participants.

For Centre 1: The total number of screens completed was 109, mostly completed at a single time point (median [IQR] 1 [1] screens). Amongst participants who commenced the CIPN-Screen, the completion rate for Screen 1 was 92.0%, and for all screens combined the completion rate was 93.6%. In terms of methods used, 84.4% of screens were completed online, and 15.6% completed on paper.

For Centre 2: The total number of screens completed was 646, mostly completed longitudinally (median [IQR] 8 [6] screens). Amongst participants who commenced the CIPN-Screen, the completion rate for Screen 1 was 97.6%, and for all screens combined the completion rate was 98.9%. All screens were completed online.

At each centre, 20–24% participants completed Screen 1 prior to neurotoxic chemotherapy commencement (Centre 1 *n* = 18, Centre 2 *n* = 17), and the remainder after commencement of neurotoxic chemotherapy.

### CIPN symptom severity by centre

**Fig. 1:**
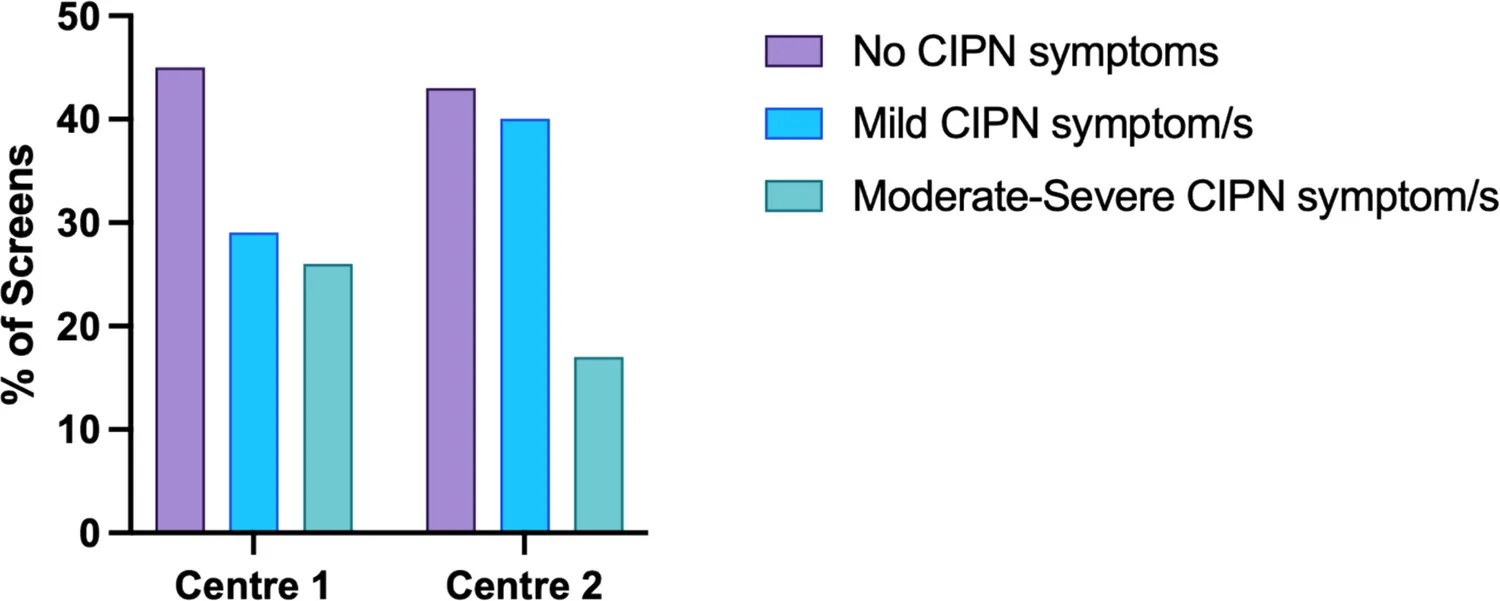
CIPN symptom severity across all screens (completed CIPN-Screen surveys) for Centre 1 and Centre 2. Bar graph illustrates the % of screens that identified No CIPN Symptom/s, Mild CIPN Symptoms, and Moderate-Severe CIPN Symptom/s across all screens for Centre 1 (*n* = 109 screens, *n* = 75 participants) and Centre 2 (*n* = 646 screens, *n* = 84 participants) on item 1 of the NCI-PRO CTCAE scale, with responses corresponding to ‘none’ plotted as No CIPN symptoms, ‘mild’ plotted as Mild CIPN symptoms and ‘moderate’, ‘severe’ or ‘very severe’ symptoms grouped and plotted as moderate-severe CIPN symptoms

Overall, 426 screens (56.4%) identified CIPN symptoms, of which 138 (18.3%) screens identified moderate to severe symptoms using the NCI-PRO-CTCAE scale. At Centre 1, a total of 60 screens (55.0%) identified CIPN symptoms and 28 (25.7%) identified moderate to severe CIPN symptoms, using item 1 of the NCI-PRO-CTCAE scale (Supplementary materials 1, Table S1; Fig. 1). At Centre 2, a total of 366 screens (56.7%) identified CIPN symptoms, with 110 (17.0%) identifying moderate to severe CIPN symptoms (Fig. 1). There was no significant difference in reported CIPN severity between Centre 1 and Centre 2 (*P* = 0.754).

At Centre 1, a total of 34 screens (31.2%) indicated that CIPN produced functional impact according to item 2 of the NCI-PRO-CTCAE. At Centre 2, 206 screens (32.1%) indicated some level of functional impact. Activities reported on the PNQ as interfered with due to therapy included sleep (Centre 1:14.4%, *n* = 24; Centre 2: 11.9%, *n* = 99), shortness of breath (Centre 1: 12.0%, *n* = 20; Centre 2: 8.8%, *n* = 73), buttoning clothes (Centre 1: 9.0%, *n* = 15; Centre 2: 8.6%, *n* = 71), working (Centre 1: 3.6%, *n* = 6; Centre 2: 11.1%, *n* = 92) and walking (Centre 1: 6.6%, *n* = 11; Centre 2: 4.7%, *n* = 39).

### Acceptability of the CIPN-Screen

Most respondents reported that the CIPN-Screen was easy to complete (92.0%, *n* = 671), easy to understand (91.6%, *n* = 668), and helpful to describe symptoms (85.6%, *n* = 624) (Supplementary materials 1, Table S2, Figure S1). There were no significant differences between centres on any of the acceptability questions (all *P* > 0.1) or on overall acceptability as measured by a composite score (*P* = 0.151).

Overall, 95.0% (*n* = 98) and 92.0% (*n* = 573) of screens at Centre 1 and Centre 2, respectively, agreed or strongly agreed that the CIPN-Screen was easy to complete. Further, 95.0% (*n* = 98) of screens at Centre 1 and 91.0% (*n* = 570) of screens at Centre 2 agreed or strongly agreed that the CIPN-Screen was easy to understand. Finally, 88.0% (*n* = 91) and 85.0% (*n* = 533) of screens at Centre 1 and Centre 2, respectively, agreed or strongly agreed that the CIPN-Screen was helpful to describe symptoms.

There was no change in acceptability over time as responses did not significantly differ between Screen 1 and 6, for: ‘I found the CIPN-Screen easy to complete’ (*P* = 0.395)*,* ‘I found the CIPN-Screen easy to understand’ (*P* = 0.879)*,* and ‘I found the CIPN-Screen helpful to assist in describing my symptoms’ (*P* = 0.772) (Supplementary materials 1, Table S3).

Acceptability composite scores were combined between centres to examine potential differences between participant groups. The composite score did not significantly differ between platinum (75 screens) and taxane-treated participants (621 screens) (*P* = 0.567); between ages <65 years (633 screens) and ≥65 years (96 screens) (*P* = 0.108); or between males (111 screens) and females (618 screens) (*P* = 0.343).

However, screens reporting none-mild symptoms reported greater acceptability (597 screens; Median [IQR] 13 [3]) than screens reporting moderate-severe symptoms (132 screens; Median [IQR] 12 [2]; *P* = 0.0002). Screens that reported moderate-severe symptoms showed a significant difference across questions (*P* = 0.01), with lower agreement for helpfulness to describe symptoms compared to other questions (Supplementary materials 1, Table S4).

### Responsiveness of CIPN-Screen to change

The screens at Centre 2 were primarily completed longitudinally (Supplementary materials 1, Figure S2). CIPN severity using the NCI-PRO-CTCAE across Screen 1–3 for Centre 2 was compared. Participants completed Screen 1 prior to (*n* = 17) or after commencement of neurotoxic chemotherapy (*n* = 67). Subsequent screens were completed following text message reminders linked to clinical visits. At Screen 1 (*n* = 84 participants), 34 (40.5%) participants reported CIPN symptoms (14 of 84 [16.7%] with moderate to severe CIPN symptoms). At Screen 2 (*n* = 76 participants), 39 (51.3%) participants identified CIPN symptoms (10 [13.2%] with moderate to severe CIPN symptoms). At Screen 3 (*n* = 72 participants), 38 (52.8%) participants identified CIPN symptoms (9 [12.5%] of these participants reported moderate to severe CIPN symptoms). A comparison of CIPN symptoms between Screen 1, 2 and 3 indicated no significant differences in symptoms between these screens (*P* = 0.515).

The changes in the CIPN symptom score over time for 52 participants that had completed Screens 1 to 7 at Centre 2 were examined. The median number of days between Screen 1 and Screen 7 was 49 [IQR 21]. At Screen 1, 14 (27%) participants reported CIPN symptoms compared to 33 (63%) participants at Screen 7. There was a significant difference across screening points (*P* < 0.0001) indicating that the CIPN-Screen detected the worsening of symptom severity over time (Supplementary materials 1, Figure S3).

### Comparison between screening tools

Individual clinical screening tools within the CIPN-Screen were compared to examine the agreement between NCI-PRO-CTCAE, PNQ and EviQ questionnaires. All screening tools were positively correlated, and for most, there were strong associations (*ρ* ≥ 0.84) (Supplementary materials 1, Table S5). The exception was the EviQ sum score which moderately (0.5–0.7) correlated with the NCI-PRO-CTCAE (*ρ* = 0.67, *P* < 0.0001) and PNQ (*ρ* = 0.66, *P* < 0.0001).

**Fig. 2:**
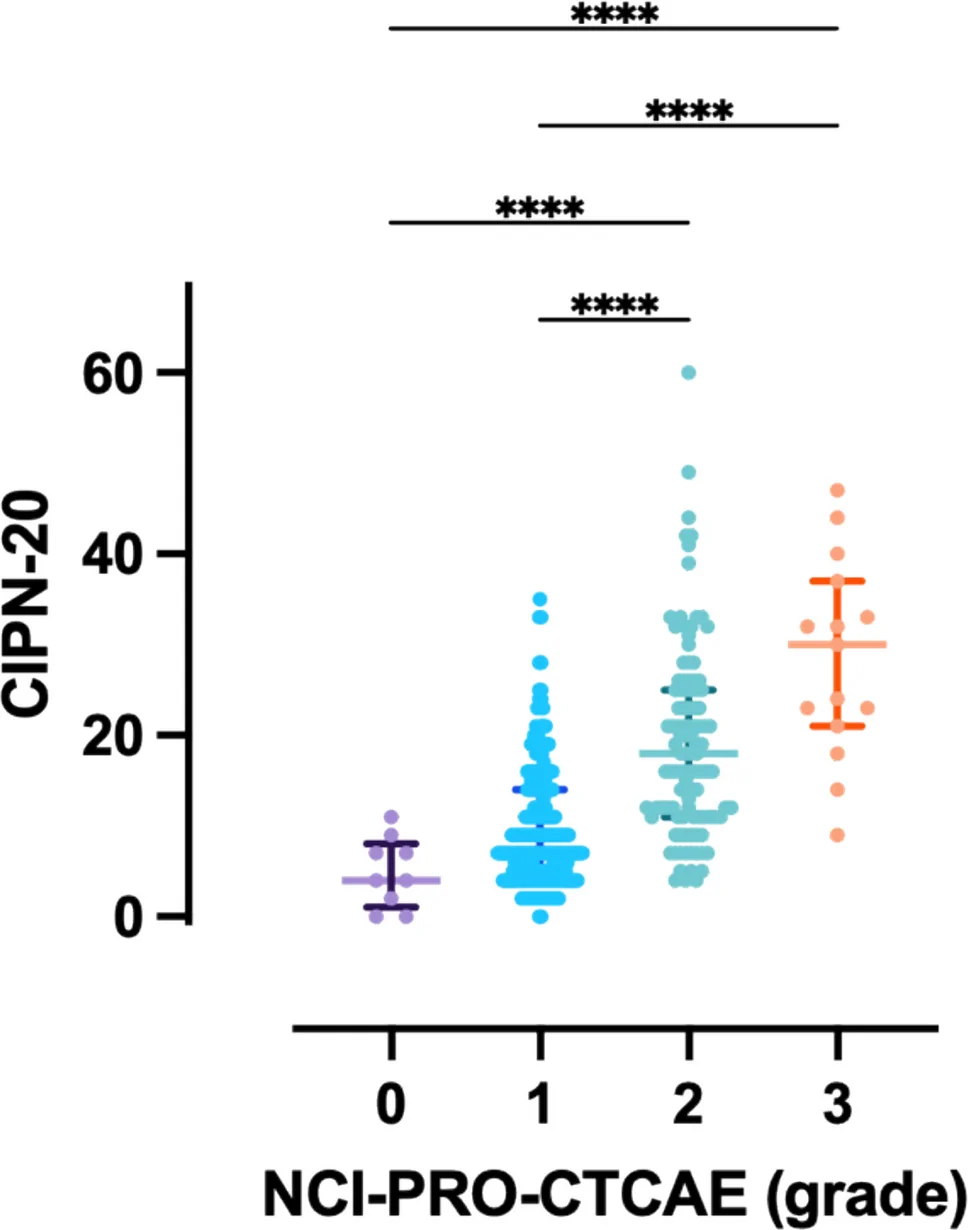
Between group comparison of the CIPN-20 and NCI-PRO-CTCAE severity grade (*n* = 381 screens). Scatterplot (median, IQR) shows a significant difference in the CIPN-20 scores across the NCI-PRO-CTCAE severity grades (Grades: 0 = none, 1 = mild, 2 = moderate, 3 = severe) (**** = *P* < 0.0001)

Participants who reported symptoms on screening tools were invited to complete the longer questionnaire EORTC-QLQ-CIPN-20. At Centre 1, 52 screens included a completed CIPN-20; the median CIPN-20 score was 11.5 [IQR 12.5]. At Centre 2, 329 screens included a completed CIPN-20; the median CIPN-20 score was 9 [IQR 12.5]. Clinical screening tools (NCI-PRO-CTCAE, PNQ, EviQ) were compared with the CIPN-20 to examine the agreement between questionnaires. All screening tools were positively correlated; however for most, these were weak (0.3–0.5) associations (NCI-PRO-CTCAE *ρ* = 0.48; PNQ *ρ* = 0.49; EviQ_sens_ *ρ* = 0.44; all *P* < 0.0001) (Supplementary materials 1, Table S5). The exception to this was the relationship between the EviQ_sum_ and CIPN-20, which demonstrated a strong positive correlation (Supplementary materials1, Table S5). Further, an intergroup comparison of the NCI-PRO-CTCAE grade and the CIPN-20 score demonstrated a significant difference between groups (*P* < 0.0001) (Fig. 2) signaling a difference in CIPN-20 score within the grades 0–3 of the NCI-PRO-CTCAE.

### Feasibility of implementation from clinician perspectives

To assess feasibility from a clinician perspective, the ORIC questionnaire was completed by 31 clinical staff (Centre 1, *n* = 13; Centre 2, *n* = 18).

At Centre 1, most respondents were medical clinicians (61.5%, *n* = 8); conversely, at Centre 2, most respondents were nursing clinicians (77.8%, *n* = 14) (Table 2). Regarding awareness of the CIPN-Screen, 46.2% (*n* = 6) and 66.7% (*n* = 12) of respondents at Centre 1 and Centre 2, respectively, indicated awareness of the CIPN-Screen study.

**Table 2:** Clinical perspectives survey participant demographics and adapted Organisational Readiness for Implementing Change (ORIC) questionnaire means and standard deviations (SD) for each Centre

	Centre 1	Centre 2
**Number of respondents**	13	18
**Role** (*n*, %)		
Senior Oncologist	8 (61.5%)	3 (16.7%)
Junior Oncologist	1 (7.7%)	-
Nurse	4 (30.8%)	14 (77.8%)
Allied Health	-	1 (5.6%)
**ORIC scores (Mean, SD)**		
ORIC total (range 12–60)	43.46 (9.96)	46.39 (9.29)
ORIC Efficacy (range 7–35)	24.23 (6.33)	26.39 (5.87)
ORIC Commitment (range 5–25)	19.23 (3.86)	20.00 (4.19)

The ORIC questionnaire contains a 5-point scale ranging from disagree (1) to agree (5), with a total score range of 12–60, with higher scores corresponding to higher levels of organisational readiness for implementation of CIPN screening. ORIC questionnaire comprises two subscales: change commitment (5 items, with a total score range of 5–25) and change efficacy (7 items, with a total score range of 7–35)

Both centres demonstrated a mean ORIC score associated with mid-range readiness for change [17] with regard to CIPN screening (Table 2; Centre 1 mean (SD) 43.46 (9.96) and Centre 2 46.39 (9.29). The two ORIC subscales change efficacy and change commitment were strongly correlated (*ρ* = 0.74, *P* < 0.0001), highlighting that individuals high in change efficacy were also high in commitment.

At both centres, the item with the highest level of agreement related to motivation and desire to implement CIPN screening (Supplementary materials 1, Table S6; Centre 1: 84.6% agreement for item 3 ‘People who work here are motivated to implement screening for chemotherapy-induced peripheral neuropathy,’ Centre 2: 88.9% agreement for item 5 ‘People who work here want to implement screening for chemotherapy-induced peripheral neuropathy’). However, the lowest level of agreement was in regard to confidence in managing the politics of implementation screening for CIPN (item 7; Centre 1: 46.2% agreement, Centre 2: 38.9% agreement).

## Discussion

This study investigated the feasibility and acceptability of implementing enhanced routine screening for CIPN across two oncology centres. Overall, most respondents at both centres agreed that the CIPN-Screen was easy to understand, easy to complete, and helpful to describe symptoms. CIPN-Screen demonstrated responsiveness to change over time. Clinicians reported some readiness to implement improved clinical screening for CIPN, highlighting the need for development of tailored implementation strategies.

### CIPN assessment approaches

Comprehensive CIPN assessment includes multiple modalities including patient-reported outcomes, neurological examination, and objective sensory testing [18]. However, for clinical screening purposes there are often additional considerations including limited time, resourcing, and implementation challenges. Additionally, in the cancer setting, there are many different symptoms and toxicities that need to be screened for and for that reason more intensive screening is not always practicable in routine clinical practice. To address this point, in this study we utilized brief PROMs for CIPN screening. CIPN PROMs are valid and responsive tools which provide information about CIPN symptoms and impact [9]. For the purposes of clinical screening, long PROM questionnaires may be perceived as too burdensome, with CIPN questionnaires with >3 items rating lower for feasibility in a Delphi consensus study [19]. Further, brief PROMs compare well to other forms of CIPN assessment, making them well-suited as routine screening tools [10].

Overall, 56.4% of screens identified CIPN symptoms, with more than 30% of symptomatic participants reporting a functional impact of CIPN on sleep, fine motor function, or mobility. These results are in keeping with prior CIPN screening studies, which reported that CIPN interfered with function in ~40% of participants [20]. There were no differences in the proportion of symptomatic participants or reported CIPN severity between centres, despite differences in participant demographics and the delivery of the CIPN-Screen between centres. In the Centre 2 cohort, the CIPN-Screen demonstrated responsiveness to change, with emergent CIPN identified when comparing screens across time, indicating that the CIPN-Screen detected symptom development over time.

### Feasibility and acceptability of the CIPN-Screen

Most respondents agreed that the CIPN-Screen was easy to understand, easy to complete, and helpful to describe symptoms. Acceptability did not significantly differ by treatment type, age group, or sex. However, interestingly we found that although acceptability was high, people with moderate to severe CIPN reported lower agreement with the statement ‘I found the CIPN-Screen helpful to assist in describing my symptoms’ compared to those without CIPN. It is possible that CIPN-Screen may not have captured the symptom range experienced by participants or that the short form PROMs provided limited opportunities for participants to describe their symptoms in detail. Further examination is required to understand this result and ensure that patients feel that their primary concerns are reported. Patients with a higher symptom burden may require greater input from clinicians in terms of CIPN management and referral to additional services, which short screening tools are not designed to assess. Additionally, a small qualitative study reported that CIPN symptoms may interfere with hand function and affect electronic PROM completion, requiring the need for modifications to support usability [21]. Further examination of the impact of CIPN symptoms on electronic symptom monitoring would be helpful to ensure that feasibility and utility is preserved across different clinical groups.

### Differences in site implementation

The CIPN-Screen was implemented differently across sites. At Centre 2, the CIPN-Screen was integrated into clinical workflow, enabling PROM reminders to be sent automatically via text message longitudinally without ongoing involvement by clinical staff, compared to Centre 1 where the method of asking participants to complete surveys longitudinally relied on clinical staff and QR Codes. This resulted in high rates of longitudinal screening at Centre 2, whereas most Centre 1 participants completed the CIPN-Screen at a single time point. Obtaining multiple screenings over the course of chemotherapy treatment is a necessary component of enhanced CIPN screening, and accordingly implementation of automated systems to remind patients through text messaging or other electronic means is critical to support uptake. However, tailored strategies will be required to ensure that use of electronic PROM collection and automated reminders do not disadvantage demographic groups who may be less able to engage with electronic data collection, as identified in prior studies [22, 23].

Ultimately, electronic PROM systems embedded in electronic medical records with full automation and within existing workflows are the only practical way to embed PROMs in routine clinical care. A further necessary step is ensuring that clinicians receive screening information in real time, to assist clinical decision-making.

### Feasibility of implementation of enhanced CIPN screening

While this study utilized multiple brief PROM screening instruments, for routine use a single instrument is likely preferred for screening, with a short PROM followed by a longer questionnaire in symptomatic patients to identify the impact of symptoms and enable triage [19]. Previous work has highlighted the reliability and validity of the PRO-CTCAE for CIPN screening [20]. The PNQ has also been identified as a highly rated screening instrument [19]. While we found strong positive correlations between different brief screening PROMs including PRO-CTCAE, PNQ, and EviQ numbness and tingling, these were often less well correlated with longer instruments, highlighting the differences in information collected.

The successful implementation of enhanced screening will also depend on the perspectives of the clinical team. The ORIC questionnaire [15] was used to examine organisational readiness. Organisational readiness incorporates both change commitment (collective determination to implement a change) and change efficacy (collective ability to implement a change). The mean ORIC scores for Centre 1 and Centre 2 were similar (43.5 and 46.4, respectively), indicating that both sites showed mid-range readiness for change, as classified in previous studies [17]. However, as organisational readiness is a critical precursor to successful change implementation, our findings suggest that this could be improved to support lasting implementation.

Implementation of patient-based symptom monitoring in routine cancer care is a global priority, with international consensus guidelines recommending the benefits of electronic symptom monitoring [24, 25]. Real-world implementation of remote symptom monitoring has been undertaken on a large scale, demonstrating high patient compliance, and clinically meaningful improvements in symptom burden [26]. However, despite these benefits, sustainable, successful implementation will require site-specific implementation strategies to support uptake. Critical features to support clinical implementation include training and supporting key staff, integrating digital systems into electronic medical records and routine clinical workflows and incorporating clinical management pathways into routine use [27, 28]. The key importance of an implementation plan was highlighted by a prior study which attempted to implement an improved CIPN care decision support algorithm [29]. The algorithm did not lead to improved assessment or management of CIPN, and the authors highlighted the need for theory-based implementation frameworks to assist with providing the basis for successful implementation [29]. Further, understanding how enhanced CIPN screening information can integrate with and assist with dosing decision making also requires further study.

### Limitations

Because this was a clinical implementation study, the method of implementation was site specific and guided by the resources and activities at each site. Consequently, there were differences between the sites in terms of implementation, which affected the amount of longitudinal screening. This is evidenced by the higher completion of screening surveys at Centre 2 compared to Centre 1. The included cohorts were restricted to patients that were approached and agreed to participate in the study, with a strong bias towards young patients with breast cancer in Centre 2 (Table 1), so we do not have data on implementation or acceptability in a broader patient group. Further, the included cohorts may not be representative of other oncology centres or populations. Additionally, we did not use an implementation framework or behaviour change approach, such as the Consolidated Framework for Implementation Research [30]. Tailoring an implementation approach on a site-specific basis is important to ensure that enhanced screening can occur within existing clinical workflows. Further planned studies will use these approaches to design more effective strategies for implementation based on these results. Further, the lack of qualitative interview data from patient or clinician participants to identify barriers and facilitators of implementation is a limitation of this study. Because the screening information was not integrated into clinical care our study was not able to determine the impact of enhanced CIPN screening on chemotherapy delivery or rates of chronic CIPN. Further, our study did not include any clinical or neurological assessment to confirm diagnosis of CIPN.

## Conclusions and next steps

These findings highlight the feasibility and acceptability of routine CIPN screening using structured PROM tools. We found that substantially more longitudinal assessments were completed when surveys were prompted by automated text messages that did not require ongoing involvement by clinical staff. This highlights the need for automation and integration of screening tools into existing workflows. Toxicity screening is a key part of supportive care in oncology, and approaches to improve the systematic delivery of patient-relevant toxicity screening are important to improve clinical care delivery and individual patient outcomes. Ultimately, the implementation of guided clinical pathways for CIPN and other toxicities and measurement of their impact are important to support quality of life and quality of care for people with cancer.

## Supplementary Information

Below is the link to the electronic supplementary material.

ESM 1 DOCX (207 KB)

## Data Availability

The datasets analysed during the current study are available from the corresponding author on reasonable request.
