# Supplementary material for: Feasibility of clinical implementation and acceptability of patient-reported screening for chemotherapy-induced peripheral neuropathy (CIPN)
Source: Support Care Cancer. 2026 Oct 8;34(11):1073. doi: 10.1007/s00520-026-11296-4 (PMC13649828; doi:10.1007/s00520-026-11296-4)
Supplement: Supplementary file 1 [file 520_2026_11296_MOESM1_ESM.docx]

**Feasibility of clinical implementation and acceptability of patient-reported screening for chemotherapy-induced peripheral neuropathy (CIPN)**

**Authors:** Alice Treloar ^1^, Peter Grimison^2,3^, Victoria Choi^2,4^, Jed Young^5^, Katelin Mayer^5^, Tiffany Li^1^, Frances Boyle^3,6^, Tracy King^7,8^, David Mizrahi^9^, Lisa G Horvath ^2,3^, David Goldstein^5,10^ Susanna B Park^1,11^

**Correspondence to**: Susanna Park, Brain and Mind Centre, The University of Sydney, Sydney, NSW, Australia. E-mail: [Susanna.Park@sydney.edu.au](mailto:Susanna.Park@sydney.edu.au)

**Supplementary Materials**

**Supplementary Methods**

**Neuropathy Screening Instruments**

The NCI-PRO-CTCAE[11] sensory neuropathy subscale consists of two items, each scored on a 5 point scale. The first item assessed severity of numbness and tingling in the hands or feet (0=none, 4=very severe, and the second item assessed the degree which these symptoms interfere with daily activities (0=not at all, 4=very much). For comparison of screening tools, a composite grade was calculated, using the grading algorithm established by Basch and colleagues[31]. The score for the severity and interference questions were applied to the algorithm matrix available in the Supplemental table S2 of Basch et al 2021 [31] to identify the composite grade which could range from 0 - 3, with higher scores denoting more severe presentations. The NCI-PRO-CTCAE is available from https://healthcaredelivery.cancer.gov/pro-ctcae/instrument-pro.html.

The PNQ[12] is a two item measure assessing sensory and motor symptom severity (0=no neuropathy, 4=severe neuropathy) and functional interference. Respondents could also specify what activities were interfered with due to neuropathy. To examine the relationship between the PNQ and the other assessments, the responses to question one in the PNQ relating to sensory symptoms and interference were used. The PNQ is available from [12].

The EviQ[13] included 11 items that assess sensory, autonomic and motor symptoms. Each item is scored on a 5-point scale ranging from none (0) to disabling/life-threatening (4). The scores for the eleven items of the EviQ were summed to calculate the EviQ sum score, which was termed EviQ_sum_ (sum scale range 0-44). In addition, the highest score from either question 1 or 2 (relating to numbness/tingling and pain) was utilised to provide a sensory symptom score, termed EviQ_sens_. The EviQ is available from https://www.eviq.org.au/clinical-resources/assessment-tools/8-anti-cancer-drug-induced-peripheral-neuropathy-s.

The EORTC-QLQ-CIPN-20[14] is a 20 item validated patient-reported outcome measure that assessed CIPN symptoms and functioning. Participants rate their symptom experience and subsequent functioning over the past week using a 4-point scale ranging from not at all (1) to very much (4). Total scores are transformed to a 0-100 scale (CIPN-20) with greater scores indicating worse neuropathy. The EORTC-QLQ-CIPN20 is available from https://qol.eortc.org/questionnaire/qlq-cipn20/.

**CIPN-Screen Acceptability**

Participants answered three acceptability questions regarding whether the CIPN-Screen was easy to complete, easy to understand and helpful to assist in describing their symptoms using a 5-point Likert scale from strongly disagree (0) to strongly agree (4). For analysis, ‘strongly disagree’ responses were coded as 1 and ‘strongly agree’ responses coded as 5, and a composite score was calculated for each screen (range 3 to 15). The acceptability questions were: 1) I found the CIPN-Screen easy to complete, 2) I found the CIPN-Screen easy to understand and 3) I found the CIPN-Screen helpful to assist in describing my symptoms.

The ORIC questionnaire[15] assesses the readiness of an organisation to implement change and comprises two subscales: change commitment (5 items) and change efficacy (7 items). The 5-point scale ranges from disagree (1) to agree (5), with a total score range of 12–60, with higher scores corresponding to higher levels of organisational readiness for implementation of CIPN screening. The ORIC questionnaire was adapted to assess the implementation of the CIPN-Screen (e.g., 'People who work here are committed to implementing screening for chemotherapy-induced peripheral neuropathy’). Clinicians and clinical care team members were also asked to indicate their role at the centre and if they were aware of the CIPN-Screen study.

**Supplementary Tables and Figures**

**Table S1.** CIPN symptom severity at Centre 1 and Centre 2. Table shows the CIPN symptom severity for all screens using item one of the NCI-PRO-CTCAE scale at Centre 1 (109 screens) and Centre 2 (646 screens). Responses corresponding to ‘none’ were classified as No CIPN symptoms, responses corresponding to either ‘mild,’ moderate’, ‘severe’ or ‘very severe’ symptoms were classified as Mild-Severe CIPN symptoms and responses corresponding to either ‘moderate’, ‘severe’ or ‘very severe’ symptoms grouped as Moderate-Severe CIPN symptoms.

|  | Centre 1 | Centre 2 |
| --- | --- | --- |
| **CIPN symptom severity for all Screens (n screens, %)** | | |
| No CIPN symptoms | 49 (45.0%) | 280 (43.3%) |
| Mild-Severe CIPN symptoms | 60 (55.0%) | 366 (56.7%) |
| Moderate-Severe CIPN symptoms | 28 (25.7%) | 110 (17.0%) |

**Table S2.** Responses to the three feasibility questions for all screens in Centre 1 (n=103 screens) and Centre 2 (n=626 screens). Participants reported their level of agreement (strongly disagree to strongly agree) to statements that the screen was easy to complete, easy to understand, and helpful in describing their symptoms

|  | **Centre 1** | **Centre 2** |
| --- | --- | --- |
| **Easy to complete (n %)** | | |
| Strongly Disagree - Disagree | 0 (0.0%) | 11 (2.0%) |
| Neutral | 5 (5.0%) | 42 (7.0%) |
| Agree – Strongly Agree | 98 (95.0%) | 573 (92.0%) |
| **Easy to understand (n, %)** | | |
| Strongly disagree - Disagree | 0 (0.0%) | 10 (2.0%) |
| Neutral | 5 (5.0%) | 46 (7.0%) |
| Agree - Strongly Agree | 98 (95.0%) | 570 (91.0%) |
| **Helpful to assist in describing symptoms (n, %)** | | |
| Strongly disagree - Disagree | 1 (1.0%) | 6 (1.0%) |
| Neutral | 11 (11.0%) | 87 (14.0%) |
| Agree – Strongly Agree | 91 (88.0%) | 533 (85.0%) |

**Table S3.** Responses to feasibility questions at Screen 1 and Screen 6 at Centre 2. Table illustrates that there was no change in feasibility responses from Screen 1 to Screen 6, as measured by the following statements: ‘I found the CIPN Screen easy to complete’ (P = 0.395), ‘I found the CIPN-Screen easy to understand’ (P = 0.879) and ‘I found the CIPN-Screen Survey helpful to assist in describing my symptoms’ (P = 0.772)

| **Centre 2** | **Screen 1 (n = 80)** | **Screen 6 (n = 58)** | **P value** |
| --- | --- | --- | --- |
| **Easy to complete (n, %)** | | | |
| Agree/Strongly Agree | 76 (95.0%) | 53 (91.4%) | *P* = 0.395 |
| Strongly Disagree/Disagree/Neutral | 4 (5.0%) | 5 (8.6%) |  |
| **Easy to understand (n, %)** | | | |
| Agree/Strongly Agree | 75 (93.8%) | 54 (93.1%) | *P* = 0.879 |
| Strongly Disagree/Disagree/Neutral | 5 (6.3%) | 4 (6.9%) |  |
| **Helpful to assist in describing symptoms (n, %)** | | | |
| Agree/Strongly Agree | 69 (86.3%) | 51 (87.9%) | *P* = 0.772 |
| Strongly Disagree/Disagree/Neutral | 11 (13.8%) | 7 (12.1%) |  |

**Table S4.** Composite feasibility responses combined for Centres 1 and 2 and compared across Questions 1, 2, and 3. Q1: ‘I found the CIPN-Screen easy to complete,’ Q2: ‘I found the CIPN-Screen easy to understand,’ and Q3: ‘I found the CIPN-Screen helpful to assist in describing my symptoms’. A Kruskal-Wallis test indicated a significant difference across Questions 1, 2 and 3 (*P* = 0.01). Further examination with a Dunn’s multiple comparison test indicated agreement was lower for Q3 compared to Q1 and Q2.

| **Moderate to Severe Symptoms (132 screens)** | **Kruskal-Wallis Test** | **Dunn’s Test** |
| --- | --- | --- |
| Q1 vs. Q2 | *P* = 0.01 | *P* > 0.99 |
| Q1 vs. Q3 |  | *P* = 0.02 |
| Q2 vs. Q3 |  | *P* = 0.04 |

**Table S5.** Bivariate Spearman correlations between the NCI-PRO-CTCAE, PNQ, EviQ_sens_, EviQ_sum_ and CIPN-20

|  | **NCI-PRO-CTCAE** | **PNQ** | **EviQ_sens_** | **EviQ_sum_** |
| --- | --- | --- | --- | --- |
| **NCI-PRO-CTCAE** | - | - | - | - |
| **PNQ** | 0.88  (n=748, *P* <0.0001) | - | - | - |
| **EviQ_sens_** | 0.84  (n=738, *P* <0.0001) | 0.85  (n=743, *P* <0.0001) | - | - |
| **EviQ_sum_** | 0.67  (n=736, *P* <0.0001) | 0.66  (n=741, *P* <0.0001) | - | - |
| **CIPN-20** | 0.48  (n=378, *P* <0.0001) | 0.49  (n=381, *P* <0.0001) | 0.44  (n=380 *P* <0.0001) | 0.76  (n=378, *P* <0.0001) |

**Table S6.** Proportion of clinical perspective survey respondents who agreed (either Agree or Somewhat Agree) to the 12-item ORIC questionnaire for Centre 1 (n = 13) and Centre 2 (n =18)

|  | **Centre 1** | **Centre 2** |
| --- | --- | --- |
| **Commitment subscale** | **Agree or Somewhat Agree** | |
| 1. People who work here are committed to implementing screening for chemotherapy-induced peripheral neuropathy. | 69.2% (n = 9) | 77.8% (n = 14) |
| 2. People who work here are determined to implement screening for chemotherapy-induced peripheral neuropathy. | 61.5% (n = 8) | 44.4% (n = 8) |
| 3. People who work here are motivated to implement screening for chemotherapy-induced peripheral neuropathy. | 84.6% (n = 11) | 83.3% (n = 15) |
| 4. People who work here will do whatever it takes to implement screening for chemotherapy-induced peripheral neuropathy. | 69.2% (n = 9) | 50.0% (n = 9) |
| 5. People who work here want to implement screening for chemotherapy-induced peripheral neuropathy. | 69.2% (n = 9) | 88.9% (n = 16) |
| **Efficacy Subscale** |  |  |
| 6. People who work here feel confident that they can keep the momentum going in implementing screening for chemotherapy-induced peripheral neuropathy. | 46.2% (n = 6) | 66.7% (n = 12) |
| 7. People who work here feel confident that they can manage the politics of implementing screening for chemotherapy-induced peripheral neuropathy. | 46.2% (n = 6) | 38.9% (n = 7) |
| 8. People who work here feel confident that the organization can support people as they adjust to screening for chemotherapy-induced peripheral neuropathy. | 46.2% (n = 6) | 83.3% (n = 15) |
| 9. People who work here feel confident that the organization can get people invested in implementing screening for chemotherapy-induced peripheral neuropathy. | 69.2% (n = 9) | 61.1% (n =11) |
| 10. People who work here feel confident that they can coordinate tasks so that implementation goes smoothly. | 61.5% (n = 8) | 66.7% (n = 12) |
| 11. People who work here feel confident that they can keep track of progress in screening for chemotherapy-induced peripheral neuropathy. | 53.8% (n = 7) | 61.1% (n = 11) |
| 12. People who work here feel confident that they can handle the challenges that might arise in implementing screening for chemotherapy-induced peripheral neuropathy. | 61.5% (n = 8) | 61.1% (n = 11) |

**Figure S1** Responses to questions relating to the feasibility of the CIPN-Screen at Centre 1 (n=103) and Centre 2 (n=626). a) Bar graph illustrates the percentage of participant agreement to the feasibility statement ‘I found the CIPN-Screen Survey easy to complete’ across all screens for Centre 1 and Centre 2. Responses did not significantly differ between centres (P=0.209). b) Bar graph shows the % participant agreement to the feasibility statement ‘I found the CIPN-Screen Survey easy to understand’ across all screens for Centre 1 and Centre 2. Responses did not significantly differ between centres (P=0.165). c) Bar graph illustrates the % of participant agreement to the feasibility statement ‘I found the CIPN-Screen Survey helpful to assist in describing my symptoms’ across all screens for Centre 1 and Centre 2. Responses did not significantly differ between centres (P=0.391)

**Figure S2** Number of screens (completed CIPN-Screen surveys) completed by patients at Centre 2 (n=646 screens, 84 patients) at each timepoint

**Figure S3** Change in CIPN symptoms over Screens (completed CIPN-Screen surveys) 1-7 for 52 patients at Centre 2. Scatterplot shows individual responses (and median and interquartile range) of symptom severity (no symptom/s, mild symptom/s, moderate symptom/s, and severe symptom/s) for screens 1-7 for 52 patients. There was a significant difference in responsiveness (P <0.0001) suggesting that the CIPN-Screen identified a change in symptoms over time
